# Effect of metformin versus metformin plus liraglutide on gonadal and metabolic profiles in overweight patients with polycystic ovary syndrome

**DOI:** 10.3389/fendo.2022.945609

**Published:** 2022-08-17

**Authors:** Chuan Xing, Han Zhao, Jiaqi Zhang, Bing He

**Affiliations:** Department of Endocrinology, Shengjing Hospital of China Medical University, Shenyang, China

**Keywords:** metformin, liraglutide, polycystic ovary syndrome, gonadal profiles, hyperandrogenemia or androgen excess

## Abstract

**Objective:**

To observe the effect of metformin (MET) monotherapy versus MET plus liraglutide (LIRA) on gonadal and metabolic profiles in overweight patients with polycystic ovary syndrome (PCOS).

**Methods:**

Sixty overweight patients with PCOS were recruited from January 2021 to January 2022 in Shengjing Hospital of China Medical University and were randomly assigned to the MET or combination (COM) group to receive 12 weeks of MET monotherapy or MET plus LIRA therapy. Anthropometric measurements, menstrual cycle changes, gonadal profiles, and oral glucose tolerance tests (OGTT) were conducted at baseline and after the 12-week treatment.

**Results:**

Fifty-two subjects completed the trial while eight were lost during the follow-up. Both MET and COM improved menstrual cycles, anthropometric parameters, and glucose metabolism after the 12-week treatment; however, there was no statistical difference between the two groups. MET plus LIRA therapy improved hyperandrogenemia, including TT (total testosterone), SHBG (sex hormone binding globulin) and FAI (free androgen index), whereas MET monotherapy only improved SHBG and FAI when compared with baseline. Furthermore, both MET monotherapy and MET plus LIRA therapy improved E2 (estradiol) while only MET plus LIRA therapy improved LH (luteinizing hormone), FSH (follicle stimulating hormone) and Prog (progesterone) more effectively than baseline. Additionally, MET plus LIRA therapy may improve TT, SHBG, FAI, LH and Prog more effectively than MET monotherapy; however, there were no significant differences on E2, FSH and LH/FSH between the two groups.

**Conclusions:**

In overweight patients with PCOS, both MET monotherapy and MET plus LIRA therapy improved glucose metabolism and relieved insulin resistance (IR). Additionally, MET plus LIRA therapy was more effective than MET monotherapy in improving reproductive abnormalities and hyperandrogenemia, potentially by modulating the hypothalamic-pituitary-ovarian axis.

## Introduction

Polycystic ovary syndrome (PCOS) is a common reproductive endocrine disease characterized by ovulatory dysfunction and hyperandrogenemia (HA), affecting 5–15% women of reproductive age (1). Meanwhile, PCOS is considered as one of the main causes of female infertility (2) with a wide range of clinical manifestations, including reproductive disorders, dermatological disorders, and metabolic abnormalities (3). Extensive clinical and epidemiological data indicates that up to 80% of women with PCOS are overweight or obese with the prevalence of abdominal obesity (4). Obesity-related insulin resistance (IR) may induce excessive luteinizing hormone (LH)-stimulated ovarian androgen production and suppress hepatic sex hormone-binding globulin (SHBG) production, thus leading to HA (5). PCOS is also characterized by elevated serum LH levels and an altered ratio between LH and the follicle stimulating hormone (FSH) (6). Women with PCOS not only have higher basal LH levels, but also exhibit an increased number of LH pulses, which together drive the synthesis of androgens by ovarian theca cells, leading to HA (7). The secretion of gonadotropin-releasing hormone (GnRH) from the brain is regulated by several upstream neural and endocrine factors that contribute to both the timing and magnitude of GnRH secretion. Increased androgen signaling in brain may be a potential mechanism in the pathophysiology of PCOS, underlying the hypersecretion of GnRH and LH (8).

Metformin (MET) is a biguanide insulin sensitizer which may reduce hepatic glucose production, stimulate insulin-mediated glucose uptake in the liver and skeletal muscle, and reduce gluconeogenic substrate utilization (9). MET is used as a second-line treatment in PCOS guidelines and has multiple beneficial effects on menstrual disturbances, ovulatory disturbances, HA, metabolic, and cardiovascular abnormalities (10, 11). Glucagon-like peptide-1 (GLP-1) is an intestinal hormone that enhances glucose-stimulated insulin secretion, inhibits glucagon secretion, delays gastric emptying, increases satiety, reduces food intake and appetite, reduces body weight, and exerts other physiological effects (12). Animal and clinical studies have revealed that the effectiveness of GLP-1 receptor agonists (GLP-1 RAs) in treating PCOS and preventing its metabolic consequences (13). Liraglutide (LIRA) is a long-acting GLP-1 analogue with 97% similarity to human GLP-1 that reduces body weight and HA while improving menstrual cycles in patients with PCOS (14). Herein, the aim of the present randomized study was to evaluate the effect of MET monotherapy versus MET plus LIRA therapy on gonadal and metabolic profiles to provide new sights for treatment of overweight patients with PCOS.

## Methods

### Patients

We recruited 60 patients aged 18 to 40 years, who were diagnosed with PCOS in the outpatient department of Endocrinology, Shengjing Hospital of China Medical University from January 2021 to January 2022. All patients were informed about the study purpose, and signed written informed consent prior to participation. This study was conducted in accordance with the Declaration of Helsinki and was approved by the Ethics Committee of Shengjing Hospital of China Medical University (registration number: 2020PS624K). The study was registered on Clinical Trials.gov (registration number: NCT04969627 with trial activation on 01/01/2021, first subject enrolled on 01/04/2021, last subject enrolled on 01/04/2022 and database lock on 03/29/2022).

The inclusion criteria for the subjects were as follows: (1) meet the Rotterdam diagnostic criteria of PCOS phenotype B with hyperandrogenism and ovulatory dysfunction (15); (2) body mass index (BMI) ≥ 24 kg/m^2^; (3) age between 18 and 40 years; (4) no medication that affects insulin sensitivity or ovarian function within the first three months of the trial; (5) use barrier contraception.

The exclusion criteria for the subjects were as follows: (1) allergy to GLP-1 RAs or MET; (2) severe cardiovascular disease; (3) abnormal liver function test results (alanine transaminase (ALT) levels 2.5 times higher than the upper limit of the normal range); (4) renal insufficiency (estimated glomerular filtration rate, eGFR < 60 mL/min/1.73 m^2^); (5) thyroid dysfunction; (6) history of cancer; (7) active infection; (8) weekly alcohol intake > 100 g; (9) pregnancy and breastfeeding; (10) 17-hydroxyprogesterone level > 2 ng/mL (to exclude women with hyperandrogenemia due to atypical 21-hydroxylase deficiency).

### Study design

**Figure 1:**
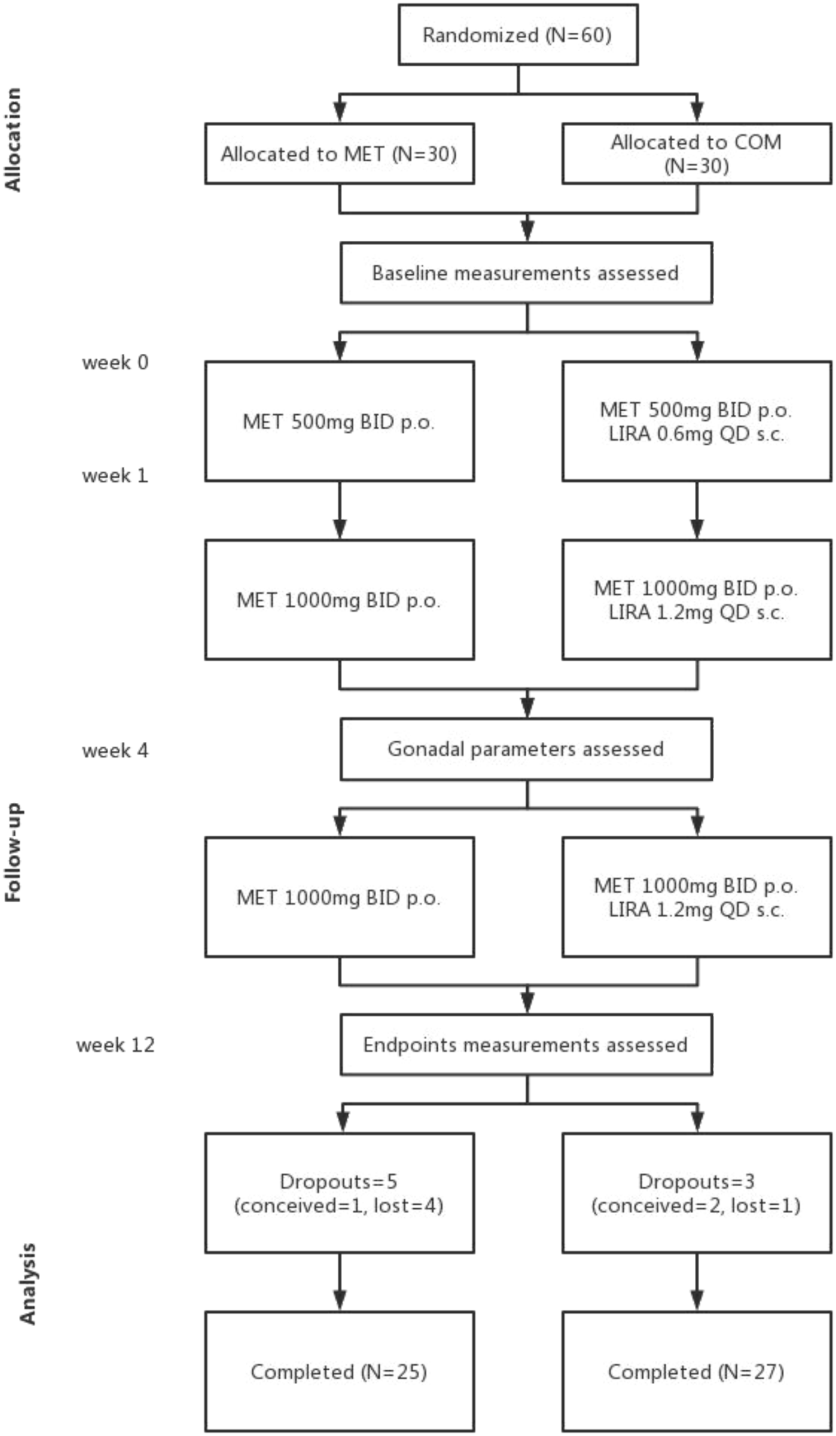
Flow chart of the study design.

This was a prospective, randomized, open-label, parallel-group controlled trial. After informed consent, the following data was obtained to determine eligibility: (1) height, weight, and age; (2) menstrual cycle; and (3) medical history. Eligible patients were assigned to the MET group or MET plus LIRA (COM) group through simple randomization at a 1:1 ratio using computer-generated codes. Patients in MET and COM group were administered MET 1000 mg BID p.o. or LIRA 1.2 mg QD s.c. plus MET 1000 mg BID p.o. for 12 consecutive weeks, respectively. Over the 12-week treatment course, all patients received the same diet and physical exercise. All subjects returned to the hospital for clinical, metabolic, and laboratory evaluations at baseline, the 4^th^ week and the 12^th^ week after treatment initiation (**Figure 1**).

### Assessment of anthropometric measures

At baseline, the 4^th^ week and the 12^th^ week after treatment, trained personnel obtained anthropometric data, including height, weight, and abdominal girth (AG) for all patients using standardized protocols. The height and weight of each subject wearing light clothes were measured to the nearest 0.1 cm and 0.1 kg, respectively. BMI was calculated as weight (kg) divided by the square of height (m). AG was measured to an accuracy of 0.1 cm by placing a tape measure around the body at the level of the navel. Women with a BMI above 24 kg/m^2^ were classified as overweight.

### Assessment of menstrual cycle

During the 12-week treatment period, patients were asked to use barrier contraception only. Menstrual cycle changes were recorded. Menstrual cycle disorders included oligomenorrhea and amenorrhea. Oligomenorrhea referred to patients with less than six menstrual periods within 12 months while amenorrhea referred to patients who have stopped menstruating for more than 6 months. Each bleeding counts as one menstrual cycle. Menstrual cycle recovery was defined as the recurrence of regular menstrual cycles in patients.

### Biochemical assessment

At baseline and the 12^th^ week after treatment, the subjects fasted for 8–12 h overnight. We collected fasting venous blood to determine fasting glucose (FG) and fasting insulin (FINS). Subsequently, we performed a 75 g oral glucose tolerance test (OGTT) and collected venous blood at 0 h, 1 h and 2 h to determine the blood glucose and insulin concentrations. The homeostasis model assessment-insulin resistance (HOMA-IR) score was calculated to evaluate IR. HOMA-IR score = FINS (mU/L) × FG (mmol/L)/22.5. The approximate trapezoidal method was used to estimate the area under the curve of glucose (AUCglu; mmol/L × h) and the area under the curve of insulin (AUCins; mU/L × h). As all patients had irregular menstrual periods at enrollment, gonadal profiles were measured when venous blood samples were collected into BD Vacutainer Tubes (SSTTM II Advance, REF 367953) at baseline, the 4^th^ and the 12^th^ week. We did not perform progestin-induced menstrual bleeding to evaluate the hormones at the follicular phase of the menstrual cycle. The samples were centrifuged at 3600 rpm and 4°C for 10 min. The serum and plasma were aliquoted and stored at –80°C before estimating serum total testosterone (TT), LH, FSH, progesterone (Prog), estradiol (E2), and SHBG levels. LH/FSH = LH (mIU/mL)/FSH (mIU/mL). Free androgen index (FAI) = TT (nmol/L)/SHBG (nmol/L) × 100. The standard glucose oxidase method was used to determine plasma glucose levels using Abbott CI16200. Radio-immunological assay was used to detect insulin, chemiluminescent immunoassay was used to detect LH, FSH, Prog, E2 and SHBG, while TT was measured by electro-chemiluminescent immunoassay using Beckman Coulter Unicel DXI800. Pre- and post-intervention samples from all subjects were analyzed in the same run, and all samples were measured in the same laboratory using standard laboratory techniques.

### Statistical analyses

The sample size was calculated using the average difference formula, assuming that the minimum average difference between the treatment groups was 20%, and the average SE (standard error) was 2.2%. The sample size required for a statistical power of 0.80 and *P*-values on both sides <0.05 was 22 per group. The final sample size was 52. The Anderson–Darling method was used to test the normality of continuous data. Continuous data were presented as mean ± standard deviation (SD) (normal distribution) or median and interquartile range (non-normal distribution). Pre- and post-treatment effects in each group were analyzed using ANOVA or paired t-test (normal distribution) or paired Wilcoxon test (non-normal distribution) if appropriate. Effects between groups at the same time point were analyzed using unpaired t-test (normal distribution) or rank test (non-normal distribution) if appropriate. Categorical variables were expressed as frequencies or percentages and compared using the chi-square test. All *P*-values were two-tailed, and *P* < 0.05 was considered statistically significant. All data analyses were performed using GraphPad Prism 8.0.1 (GraphPad Software, Chicago, IL, USA) and SPSS 23.0 (SPSS Inc., Chicago, IL, USA).

## Results

### Participants

Sixty participants were randomly allocated to receive MET 1000 mg BID p.o. monotherapy (MET group) or MET 1000 mg BID p.o. plus LIRA 1.2 mg QD s.c. therapy (COM group). For all patients receiving MET, the dose began at 500 mg BID and gradually reached the target dose of 1000 mg BID after 1 week. For all patients receiving LIRA, the dose began at 0.6 mg QD and gradually reached the target dose of 1.2 mg QD after 1 week. Only 52 participants (25 in the MET group and 27 in the COM group) completed the 12-week follow-up; three participants conceived (one in the MET group and two in the COM group), and five participants could not be contacted at the end of the 12^th^ week (four in the MET group and one in the COM group). Mild gastrointestinal side effects, such as nausea, heartburn, vomiting, and diarrhea, occurred in both groups during the first two weeks of treatment with a higher proportion of these adverse reactions in the COM group. Two participants had one episode of hypoglycemia, while one participant in the COM group developed a rash at the injection site. Most adverse reactions were mild and spontaneously resolved after 2 weeks of treatment. No participants dropped out owing to drug intolerance in both groups with the final compliance 83.3% in the MET group and 90% in the COM group. All 52 subjects underwent anthropometric measurements, menstruation monitoring, and gonadal profiles detection at baseline, the 4^th^ and the 12^th^ week; however, only 38 subjects (16 in the MET group and 22 in the COM group) underwent OGTT tests at baseline and the 12^th^ week (**Figure 1**). All subjects ranged in age from 16 to 32 years (mean ± SD, 24.73 ± 4.65 years) with an average BMI of 29.26 ± 3.84 kg/m^2^, and anovulation (menstrual cycle delay of longer than 2 months) at baseline.

### Baseline results

The characteristics of the subjects at baseline are presented in **Table 1**. There was no significant difference between the two groups on Anthropometric parameters (weight, BMI, and AG), menstrual cycle recovery rates, gonadal parameters (E2, LH, FSH, LH/FSH, Prog, TT, SHBG, and FAI), and metabolic parameters (FG, FINS, HOMA-IR, AUCglu, AUCins, and AUCglu/AUCins) at baseline.

**Table 1:** Menstruation and anthropometric, gonadal, and metabolic parameters at baseline.

	MET (N = 25)	COM (N = 27)	*P*
Age (years)	23.52 ± 4.65	25.85 ± 4.45	0.071
Regular menstrual cycles (%,n)	0, 0	0, 0	0.999
Weight (kg)	76.50 ± 12.44	79.09 ± 8.46	0.381
BMI (kg/m^2^)	28.80 ± 4.25	29.69 ± 3.44	0.411
AG (cm)	91.96 ± 12.09	95.69 ± 8.94	0.210
FG (mmol/L)	5.48 ± 0.52	5.70 ± 0.93	0.373
FINS (μU/mL)	18.13 ± 11.73	18.89 ± 7.15	0.820
HOMA-IR	4.53 ± 3.07	4.85 ± 2.09	0.708
AUCglu (mmol/L*min)	1009.00 ± 109.00	1135.00 ± 144.50	0.264
AUCins (mU/L*min)	13,305.00 ± 4458.00	13,223.00 ± 3817.00	0.960
AUCins/AUCglu	11.31 ± 7.17	11.22 ± 7.11	0.970
E2 (pg/mL)	35.00 (27.49–40.50)	46.00 (35.33–66.50)	0.062
LH (mIU/mL)	11.97 ± 4.20	12.09 ± 5.26	0.925
FSH (mIU/mL)	6.03 ± 1.48	6.22 ± 1.44	0.652
LH/FSH	2.12 ± 0.97	2.04 ± 0.98	0.781
PRL (ng/mL)	10.81 ± 4.21	10.21 ± 3.66	0.583
Prog (ng/mL)	0.55 ± 0.29	0.42 ± 0.21	0.054
TT (ng/mL)	0.84 ± 0.25	0.79 ± 0.20	0.408
SHBG (nmol/L)	18.10 (11.75–24.10)	18.80 (14.90–21.90)	0.713
FAI (%)	15.62 (10.99–26.53)	13.94 (11.93–19.40)	0.537

BMI, body mass index; AG, abdominal girth; FG, fasting glucose; FINS, fasting insulin; HOMA-IR, homeostasis model assessment-insulin resistance; AUCglu, area under the curve (AUC) for glucose; AUCins, AUC for insulin; E2, estradiol; LH, luteinizing hormone; FSH, follicle-stimulating hormone; PRL, prolactin; Prog, progesterone; TT, total testosterone; SHBG, sex hormone-binding globulin; FAI, free androgen index. Results are expressed as mean ± SD or median (25^th^–75^th^ percentile).

### Menstruation and anthropometric measurements after treatment

**Figure 2:**
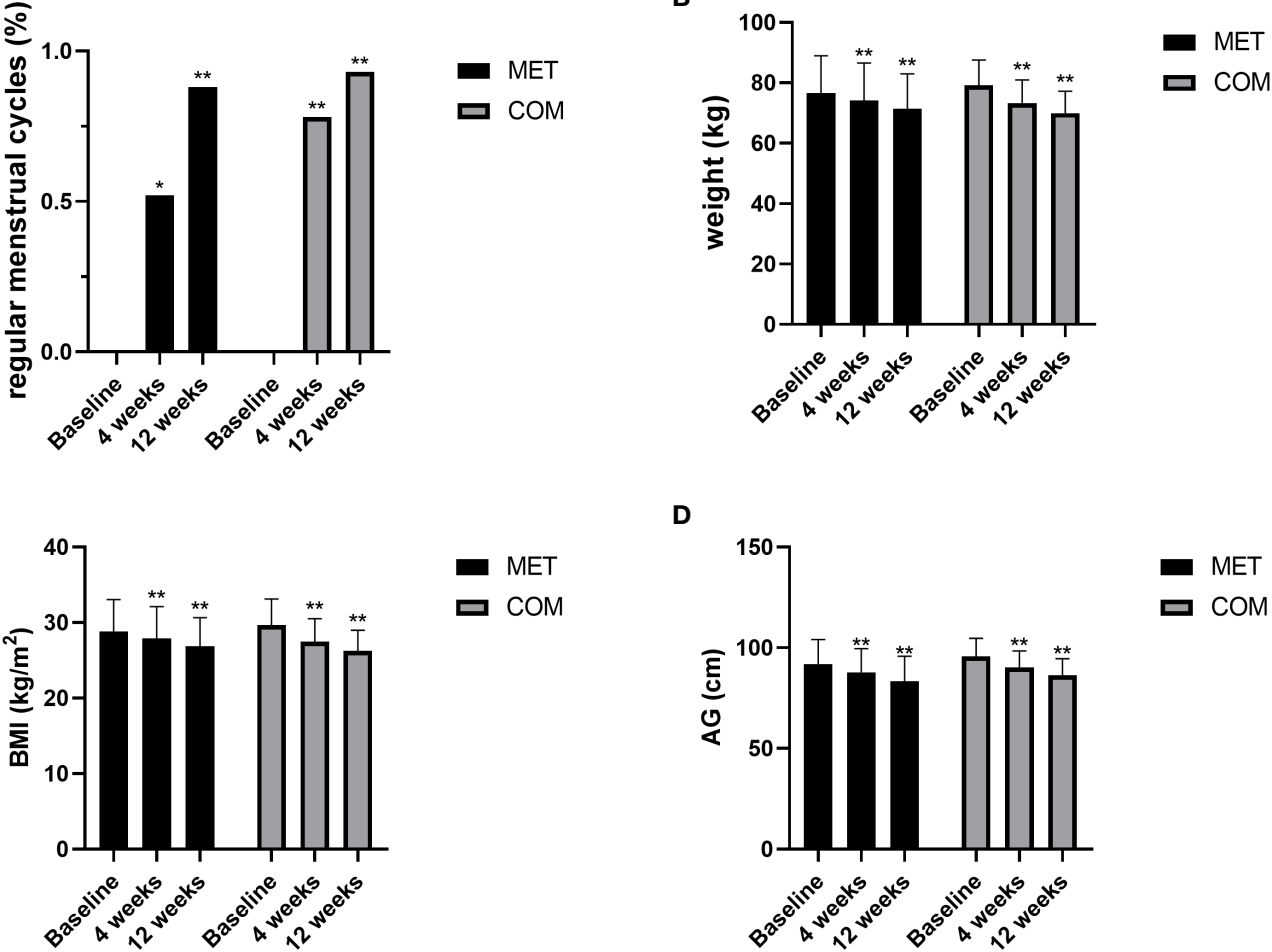
Changes in menstruation and anthropometric measurements after MET and COM therapy. **(A)** Changes in menstrual cycles after MET and COM therapy. **(B)** Changes in weight after MET and COM therapy. **(C)** Changes in BMI after MET and COM therapy. **(D)** Changes in AG after MET and COM therapy. MET, metformin; COM, combine; BMI, body mass index; AG, abdominal girth. Results are expressed as mean ± SD; ^*^ *P* < 0.05, ^**^ *P* < 0.01 (vs. before treatment in each group).

After the 4-week treatment, the recovery rate of menstrual cycle was 52.00% (13/25) in the MET group (*P* < 0.05) and 77.78% (21/27) in the COM group (*P* < 0.01). After the 12-week treatment, the recovery rate of menstrual cycle was 88.00% (22/25) in the MET group (*P* < 0.01) and 92.59% (25/27) in the COM group (*P* < 0.01). There was no difference between the two groups (**Figure 2A** and **Table 2**).

**Table 2:** Changes of hormonal, metabolic and anthropometric parameters.

Parameters	MET (N=25)			COM (N=27)			*^a^P*	*^b^P*
	Baseline	4 weeks	12 weeks	Baseline	4 weeks	12 weeks		
Regular menstrual cycles (%,n)	0%,0	52.00%,13^*^	88.00%,22^**^	0%,0	77.78%,21^**^	92.59%,25^**^	0.08	0.662
Weight (kg)	76.50 ± 12.44	74.10 ± 12.45^**^	71.44 ± 11.50^**^	79.09 ± 8.46	73.26 ± 7.71^**^	69.96 ± 7.23^**^	0.773	0.586
BMI (kg/m2)	28.80 ± 4.25	27.89 ± 4.21^**^	26.88 ± 3.76^**^	29.69 ± 3.44	27.49 ± 3.03^**^	26.24± 2.75^**^	0.69	0.485
AG (cm)	91.96 ± 12.09	87.82 ± 11.79^**^	83.40 ± 12.42^**^	95.69 ± 8.94	90.28 ± 8.20^**^	86.30 ± 8.25^**^	0.384	0.331
FG (mmol/L)	5.48 ± 0.52		5.20 ± 0.32^*^	5.70 ± 0.93		5.05 ± 0.40^**^		0.222
FINS (μU/mL)	18.13 ± 11.73		12.87 ± 7.93^**^	18.89 ± 7.15		11.60 ± 4.22^**^		0.577
HOMA-IR	4.53 ± 3.07		2.86 ± 2.05^**^	4.85 ± 2.09		2.62 ± 1.05^**^		0.677
AUCglu (mmol/L*min)	1009.00 ± 109.00		1007.00 ± 89.43	1135.00 ± 144.50		972.70 ± 135.70		0.687
AUCins (mU/L*min)	13305.00 ± 4458.00		11820.00 ± 3867.00	13223.00 ± 3817.00		8964.00 ± 2919.00		0.253
AUCins/AUCglu	11.31 ± 7.17		10.14 ± 6.84	11.22 ± 7.11		8.39 ± 5.50		0.387
E2 (pg/mL)	35.00 (27.49-40.50)	46.00 (35.33-66.50)^*^	71.10 (48.37-120.00)^**^	40.00 (32.00-67.00)	54.00 (41.00-99.00)^**^	90.00 (58.00-184.00)^**^	0.053	0.181
LH (mIU/mL)	11.97 ± 4.20	12.08 ± 6.47	9.77 ± 5.81	12.09 ± 5.26	9.54 ± 4.52	6.61 ± 4.72^**^	0.105	***0.036***
FSH (mIU/mL)	6.03 ± 1.48	5.91 ± 1.72	5.28 ± 2.11	6.22 ± 1.44	5.15 ± 1.93^**^	4.42 ± 2.59^**^	0.14	0.197
LH/FSH	2.12 ± 0.97	2.03 ± 1.02	1.91 ± 0.86	2.04 ± 0.98	1.94 ± 0.80	1.59 ± 0.95	0.725	0.215
PRL (ng/mL)	10.81 ± 4.21	10.80 ± 4.52	13.07 ± 5.77^*^	10.21 ± 3.66	10.65 ± 4.64	12.91 ± 4.97^**^	0.903	0.914
Prog (ng/mL)	0.50 (0.29-0.75)	0.45 (0.29-1.14)	0.54 (0.31-1.72)	0.42 (0.22-0.56)	0.53 (0.39-1.21)	1.08 (0.52-10.61)^**^	0.272	***0.020***
TT (ng/mL)	0.84 ± 0.25	0.81 ± 0.35	0.79 ± 0.31	0.79 ± 0.20	0.70 ± 0.24	0.62 ± 0.24^**^	0.163	***0.032***
SHBG (nmol/L)	18.10 (11.75-24.10)	19.90 (13.90-27.45)	22.40 (15.25-34.60)^**^	18.80 (14.90-21.90)	22.10 (17.10-26.90)^*^	27.00 (22.60-44.90)^**^	0.395	***0.018***
FAI (%)	15.62 (10.99-26.53)	14.22 (6.85-21.85)	12.72 (6.40-17.71)^*^	13.94 (11.93-19.40)	9.55 (5.78-17.01)	7.06 (3.90-10.19)^**^	0.104	***0.004***

BMI, body mass index; AG, abdominal girth; FG, fasting glucose; FINS, fasting insulin; HOMA-IR, homeostasis model assessment-insulin resistance; AUCglu, area under the curve (AUC) for glucose; AUCins, AUC for insulin; E2, estradiol; LH, luteinizing hormone; FSH, follicle-stimulating hormone; PRL, prolactin; Prog, progesterone; TT, total testosterone; SHBG, sex hormone-binding globulin; FAI, free androgen index. Results are expressed as mean ± SD or median (25^th^–75^th^ percentile). ^*^P < 0.05, post-treatment vs. baseline; ^**^P < 0.01, post-treatment vs. baseline; ^a^P < 0.01, COM vs. MET at the 4^th^ week; ^b^P < 0.01, COM vs. MET at the 12^th^ week. Statistically significant results are presented in bold.

After the 4-week and 12-week treatment, body weight was significantly decreased when compared with baseline in both the MET group (*P* < 0.01) and the COM group (*P* < 0.01). After the 4-week and 12-week treatment, BMI was significantly decreased when compared with baseline in both the MET group (*P* < 0.01) and the COM group (*P* < 0.01). AG was also significantly decreased when compared with baseline after the 4-week and 12-week treatment in both the MET group (*P* < 0.01) and the COM group (*P* < 0.01). There was no significant difference between the two groups in improving body weight, BMI, and AG (**Figures 2B–D** and **Table 2**).

### Metabolic parameters after treatment

**Figure 3:**
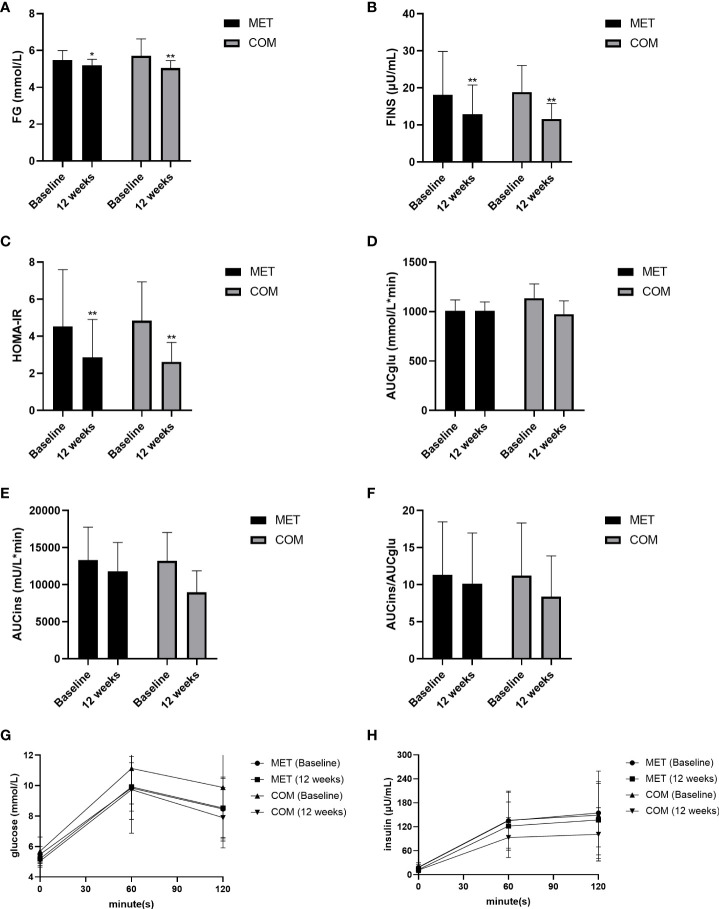
Changes in metabolic parameters after MET and COM therapy. **(A)** Changes in FG after MET and COM therapy. **(B)** Changes in FINS after MET and COM therapy. **(C)** Changes in HOMA-IR after MET and COM therapy. **(D)** Changes in AUCglu after MET and COM therapy. **(E)** Changes in AUCins after MET and COM therapy. **(F)** Changes in AUCglu/AUCins after MET and COM therapy. **(G)** Changes in OGTT (glucose) after MET and COM therapy. **(H)** Changes in OGTT (insulin) after MET and COM therapy. MET, metformin; COM, combine; FG, fasting glucose; FINS, fasting insulin; HOMA-IR, homeostasis model assessment-insulin resistance; AUCglu, area under the curve (AUC) for glucose; AUCins, AUC for insulin. Results are expressed as mean ± SD; ^*^ *P* < 0.05, ^**^ *P* < 0.01 (vs. before treatment in each group).

After the 12-week treatment, FG, FINS, and HOMA-IR were significantly decreased in both the MET group (*P* < 0.05) and the COM group (*P* < 0.01). There was no significant difference between the two groups in improving FG, FINS, and HOMA-IR (**Figures 3A–C** and **Table 2**). However, AUCglu, AUCins, and AUCins/AUCglu were not improved in both groups after the 12-week treatment (**Figures 3D–H** and **Table 2**).

### Gonadal parameters after treatment

**Figure 4:**
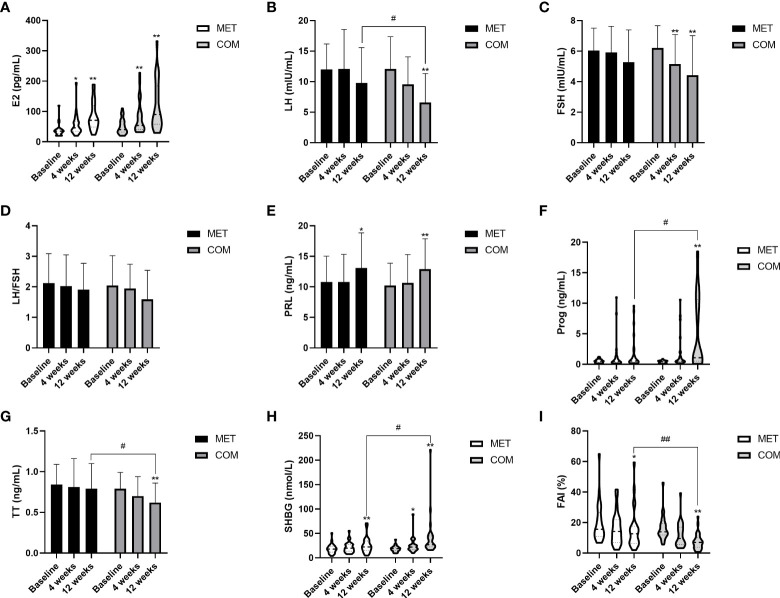
Changes in gonadal parameters after MET and COM therapy. **(A)** Changes in E2 after MET and COM therapy. **(B)** Changes in LH after MET and COM therapy. **(C)** Changes in FSH after MET and COM therapy. **(D)** Changes in LH/FSH after MET and COM therapy. **(E)** Changes in PRL after MET and COM therapy. **(F)** Changes in Prog after MET and COM therapy. **(G)** Changes in TT after MET and COM therapy. **(H)** Changes in SHBG after MET and COM therapy. **(I)** Changes in FAI after MET and COM therapy. MET, metformin; COM, combine; E2, estradiol; LH, luteinizing hormone; FSH, follicle-stimulating hormone; PRL, prolactin; Prog, progesterone; TT, total testosterone; SHBG, sex hormone-binding globulin; FAI, free androgen index. Results are expressed as mean ± SD or median (25^th^–75^th^ percentile); ^*^ *P* < 0.05, ^**^ *P* < 0.01 (vs. before treatment in each group); ^#^ *P* < 0.05, ^##^ *P* < 0.01 (vs. the other treatment).

E2 increased when compared with baseline in both the MET group (*P* < 0.05) and COM group (*P* < 0.01) (**Figure 4A** and **Table 2**). SHBG was higher than baseline only after the 12-week treatment in the MET group (*P* < 0.01) while it was higher than baseline after both the 4-week and the 12-week treatment in the COM group (*P* < 0.01) (**Figure 4H** and **Table 2**). After the 12-week treatment, FAI decreased significantly in both the MET group (*P* < 0.01) and the COM group (*P* < 0.01) (**Figure 4I** and **Table 2**). After the 12-week treatment, LH (*P* < 0.01), FSH (*P* < 0.01), Prog (*P* < 0.01), and TT (*P* < 0.01) improved significantly only in the COM group (**Figures 4B, C, F, G** and **Table 2**). After the 4-week treatment, a significant decrease from baseline in FSH (*P* < 0.05) was observed only in the COM group (**Figure 4C** and **Table 2**). However, there was no significant difference in LH/FSH between the two groups after the 4-week and 12-week treatment (**Figure 4D** and **Table 2**). After the 12-week treatment, LH [(9.77 ± 5.81) mIU/mL vs. (6.61 ± 4.72) mIU/mL, *P* = 0.036], Prog [0.54 (0.31–1.72) ng/mL vs. 1.08 (0.52–10.61) ng/mL, *P* = 0.020], TT [(0.79 ± 0.31) ng/mL vs. (0.62 ± 0.24) ng/mL, *P* = 0.032], SHBG [22.40 (15.25–34.60) nmol/L vs. 27.00 (22.60–44.90) nmol/L,*P* = 0.018], and FAI [12.72 (6.40–17.71)% vs. 7.06 (3.90–10.19)%, *P* = 0.004] were significantly improved, and more improvement was observed in the COM group. There was no significant difference between the two groups in improving E2, FSH and LH/FSH (**Figures 4A–I** and **Table 2**).

## Discussion

This study was the first randomized controlled trial designed to compare the effect of MET monotherapy versus MET plus LIRA therapy mainly focusing on gonadal profiles in overweight patients with PCOS. Our results demonstrated that both MET monotherapy and MET plus LIRA therapy improved E2, SHBG and FAI, whereas only MET plus LIRA therapy improved LH, FSH, Prog and TT more effectively than baseline. Additionally, MET plus LIRA therapy was more effective than MET monotherapy in improving serum LH, Prog, TT, SHBG, and FAI levels after the 12-week treatment. We also found that both MET plus LIRA therapy and MET monotherapy improved menstrual cycles, anthropometric parameters, and glucose metabolism with no significant differences between the two groups.

Approximately 98% of women with PCOS experienced menstrual disorders and infertility (16). Sever et al. found no statistically significant changes after the 12-week intervention in menstruation frequency, neither over time nor when analyzing separately by the type of therapy among LIRA monotherapy, MET monotherapy, or COM therapy groups (17). However, our results showed that menstrual rates were significantly improved in both the MET (88.00%) and COM (92.59%) groups after 12 weeks of treatment with no significant difference between the two groups. The difference between our results and those of Sever et al. may be due to the fact that all of our subjects initially had amenorrhea for more than 3 months and we focused on menstrual recovery, while Sever et al. focused on changes in menstrual frequency. In addition, there was great heterogeneity due to the small number of participants and the short duration of the intervention. So far, few clinical studies have observed the effect of MET and LIRA on menstrual changes in PCOS. Thus, more research is needed in the future to further clarify their effects.

Approximately 60–70% of patients with PCOS are obese, and they have greater AG than women who are simply obese (18). Reductions in BMI, particularly abdominal fat, may play an important role in reducing infertility risk factors, leading to improvements in HA and clinical symptoms (19). In addition to lifestyle intervention and combined oral contraceptives, MET is the most frequent treatment modality in PCOS to relieve IR. However, MET does n0t substantially reduce weight or alter fat distribution in PCOS. In contrast, Frøssing et al. demonstrated that 1.8 mg QD LIRA treatment for 26 weeks could achieve a substantial reduction in liver fat content by 44% and visceral adipose tissue by 18%, with a minor reduction in total body fat, thus leading to weight loss by 5.2 kg (5.6%) and a reduction in the prevalence of the nonalcoholic fatty liver disease by two-thirds when compared with placebo (20). Sever et al. found that after the 12-week treatment, the COM therapy of MET 1000 mg BID and LIRA 1.2 mg QD was superior to either LIRA 1.2 mg QD monotherapy or MET 1000 mg BID monotherapy in reducing weight, BMI, and waist circumference. Subjects in the COM group lost on average of 6.5 ± 2.8 kg compared with a 3.8 ± 3.7 kg loss in the LIRA group and a 1.2 ± 1.4 kg loss in the MET group. BMI decreased by 2.4 ± 1.0 in the COM group compared with 1.3 ± 1.3 in the LIRA group and 0.5 ± 0.5 in the MET group. Waist circumference also decreased by 5.5 ± 3.8 cm in the COM group compared with 3.2 ± 2.9 cm in the LIRA group and 1.6 ± 2.9 cm in the MET group (17). Jensterle et al. found that BMI and waist circumference reduction in the LIRA 3 mg QD monotherapy group was greater than that in the COM therapy of MET 1000 mg BID and LIRA 1.2 mg QD group (−2.2 ± 1.3 vs −1.3 ± 0.9 kg/m^2^ and −4.2 ± 3.4 vs −2.2 ± 6.2 cm) within 12 weeks (21). We found that body weight, BMI, and AG were significantly decreased when compared with baseline in both the MET and the COM groups after 4 and 12 weeks of treatment; however, no statistical differences between the two groups were found. This finding was possibly due to the short duration of the intervention or the different dosage of interventions or the initial BMI of our Chinese participants was lower than that of the European participants included by Sever et al. and Jensterle et al.

MET inhibits the gluconeogenesis of hepatic glycogen, increases glucose uptake and utilization by peripheral tissues, improves hepatic insulin sensitivity and FG in patients with PCOS (22). GLP-1 improves hepatic IR, increases hepatic glucose uptake, and improves adipose IR, thereby reducing free fatty acid production (23). GLP-1 RAs activate GLP-1 receptors on beta cells, mimicking endogenous GLP-1 to stimulate glucose-dependent phase 1 and 2 insulin secretion, thereby reducing fasting and postprandial blood glucose (24). Frøssing et al. demonstrated that the 26-week LIRA treatment caused significant reductions in FG −0.24 [−0.44 to −0.04] mmol/L, HbA1c −1.38 [−2.48 to −0.28] mmol/mol, and AUCglu when compared with placebo, whereas HOMA-IR and AUCins remained unchanged (20). Additionally, Sever et al. found that HOMA-IR tended to be reduced after LIRA monotherapy, MET monotherapy, and COM therapy, but did not significantly decrease in any group while FG and FINS did not consistently improve either after the 12-week intervention. However, patients in the COM group proved the most successful at reducing glucose value after 120 min during OGTT (17). Moreover, Jensterle et al. found that the beneficial effects on glucose metabolism after a 12-week intervention were comparable in both LIRA 3 mg QD monotherapy group and the COM therapy of MET 1000 mg BID and LIRA 1.2 mg QD group (21). Similarly, our results showed that FG, FINS, and HOMA-IR were significantly decreased in both the MET and the COM groups after 12 weeks of treatment with no significant differences between the two groups. However, AUCglu, AUCins, and AUCins/AUCglu were not significantly improved in either group after 12 weeks of treatment. The different results of glucose metabolism changes obtained in different studies may be due to the different drug dosage or intervention duration, which still need to be further confirmed by larger-scale studies in the future.

HA is the most common hormonal change in PCOS, and women with PCOS typically present elevated serum levels of multiple androgens (25). Chen et al. found that women with PCOS have higher LH levels and lower FSH levels when compared with healthy women (26). Although the LH/FSH ratio is not a diagnostic criterion of PCOS, patients with LH/FSH > 2 may have increased adrenal androgen activity and HA, which may lead to the worsening of PCOS symptoms such as hirsutism and acne (27). The ovarian phenotype in PCOS is due to maturation arrest of FSH-sensitive follicles, thus preventing them from reaching full size typically due to IR. This leads to elevated levels of insulin and insulin-related growth factors, which stimulate theca cells to produce large amounts of androgens, and in turn interfere with follicle growth, causing abnormal ovulation (28). Patients with PCOS demonstrate increased levels of GnRH leading to a higher frequency of LH pulsation, stimulation of LH-mediated androgen production, and disruption of follicle development. The resulting chronic anovulation is due to the relatively low level of FSH that occurs secondary to the altered GnRH release pattern (29). Recent studies in induced rat models have shown the expression of GLP receptors in the hypothalamus, pituitary, the change of ovary during the ovulatory cycle. Treatment with natural GLP-1 quadrupled the amplitude of the LH surge and resulted in higher progesterone in the luteal phase (30). Based on research regarding the impact of GLP-1RAs on the LH ratio, Bednarz et al. advocated that its modulating abilities can either increase LH surge in hypothalamic-pituitary-ovarian (HPO) axis disturbances due to adipose tissue estrogen aromatization or decrease excessive LH levels that induced ovarian androgen secretion which may also be related to hyperinsulinemia (31). Although trials conducted on GLP-1 RAs in PCOS have all demonstrated improvement in glucose and metabolic parameters, limited clinical research has been performed on gonadal profiles. Our study observed for the first time that COM therapy was more effective than MET monotherapy in reducing serum LH levels [(9.77 ± 5.81) mIU/mL vs. (6.61 ± 4.72) mIU/mL, *P* = 0.036] after 12 weeks of treatment. In addition, COM therapy was more effective than MET monotherapy in increasing serum Prog levels [0.54 (0.31–1.72) ng/mL vs. 1.08 (0.52–10.61) ng/mL, *P* = 0.020] after 12 weeks of treatment, indicating the recovery of local hyperandrogenic state and ovulatory function of PCOS ovaries.

Women with PCOS typically have low serum SHBG levels owing to IR and obesity, thus resulting in high levels of bioavailable active free testosterone (FT), leading to higher rates of menstrual disorders, ovarian cysts, hypo-ovulation, infertility, and hirsutism. Therefore, both the decreased circulating insulin and improved IR status can cause the increase of serum SHBG levels, leading to the decreased serum FT levels, thereby improving outcomes in patients with PCOS (27). Nylander et al. reported that after 26 weeks of LIRA treatment, SHBG levels increased by 7.4 nmol/L (95% CI 4.1 to 10.7), FT levels decreased by 0.005 nmol/L (95% CI −0.009 to −0.001), while TT levels remained unchanged (32). In a 12-week study, Jensterle et al. found that both LIRA and LIRA plus MET therapy resulted in increased SHBG levels and decreased FT levels with no difference between groups in obese patients with PCOS. However, TT levels decreased only after LIRA plus MET therapy (33). Our 12-week study revealed that SHBG levels were significantly increased and FAI significantly decreased in both MET and COM groups. Moreover, the TT level remained unchanged after 12 weeks of treatment in the MET group but significantly decreased in the COM group, which was also consistent with the findings by Jensterle et al. (33). After 12 weeks of treatment, the COM group had significant differences in the serum TT levels [(0.79 ± 0.31) ng/mL vs. (0.62 ± 0.24) ng/mL, *P* = 0.032], serum SHBG levels [22.40 (15.25–34.60) nmol/L vs. 27.00 (22.60–44.90) nmol/L, *P* = 0.018], and FAI [12.72 (6.40–17.71)% vs. 7.06 (3.90–10.19)%, *P* = 0.004] when compared with those in the MET group, indicating that MET plus LIRA therapy may be more effective than MET monotherapy in improving HA in overweight patients with PCOS.

PCOS is a heterogeneous disease with multiple factors influencing its treatment. To date, several therapeutic strategies have been proposed for the treatment of PCOS, anti-androgens for menstrual disorders and hirsutism/acne; ovulation-stimulating agents for infertility; insulin-sensitizing compounds for hyperinsulinemia. Additionally, lifestyle changes, such as diet and exercise, are considered first-line treatments for women affected by PCOS. Recent evidence also suggests that a low-carbohydrate, ketogenic diet can have beneficial effects on weight loss and improved insulin resistance in 11 women with PCOS with a BMI >27 kg/m^2^ following a 24-week low-ketogenic diet. Preliminary studies have reported significant improvements in body weight, free testosterone, LH/FSH ratio, and FINS (34). Different forms of fasting, such as intermittent fasting and regular fasting, can lower glucose and insulin levels, which can have beneficial effects on ovarian function, androgen excess, and infertility in women with PCOS. Fasting may also improve symptoms and signs associated with hyperandrogenemia. In addition, weight loss reduces adipose tissue and may negatively regulate androgen conversion in estrone: in this case, fasting may reduce hypothalamic and pituitary dysregulation, which underlie subfertility in women with PCOS (35). Inositol is a polyol with nine naturally occurring stereoisomers, including D-chiro-inositol (DCI) and inositol (MI), which play important roles in the metabolism of glucose and free fatty acids. Moreover, MI and DCI have been classified as insulin sensitizers and appear to adequately counteract several IR-related metabolic alterations with safe nutritional profiles (36). Laganà et al. administered MI or DCI to two different groups of PCOS patients and found that circulating androgen levels were decreased, LH and LH/FSH ratios were decreased in both groups, through a decrease in the HOMA index, and an increase in SHBG. The results suggest that MI appears to have the most significant effect on the metabolic profile, whereas DCI mainly affects hyperandrogenemia parameters. Furthermore, both groups showed improved menstrual cycle regularity without any significant difference between the two inositol isoforms (37). Unfortunately, our clinical trial only explored the effects of MET and LIRA in overweight PCOS patients, so future longitudinal cohort studies, as well as prospective intervention trials, may help to better elucidate the role of different treatments for PCOS.

The main limitations of our study are the single-center design, relatively small sample size, and short treatment duration. Above all, the 12-week period is very short to compare the sustainability of the benefits of these regimens. Although LIRA plus MET therapy was more effective than MET monotherapy in improving LH, Prog, SHBG, and FAI, longer treatment durations and larger, multicenter, multiethnic group trials are needed to confirm our findings. Additionally, at the end of the 12^th^ week of treatment, some patients did not undergo OGTT tests, resulting in fewer available results for glucose metabolism analysis. The lack of data hindered comprehensive evaluation of the dynamics underlying glucose metabolism. Finally, owing to various reasons, such as the COVID-19 pandemic, the rate of loss of participants during follow-up was relatively high. Although attrition was within the generally accepted threshold of 20%, it might cause potential biases.

## Conclusions

In overweight Chinese patients with PCOS, both MET monotherapy and MET plus LIRA therapy improved glucose metabolism and relieved IR. Additionally, MET plus LIRA therapy was more effective than MET monotherapy in improving reproductive abnormalities and hyperandrogenemia, potentially by modulating the hypothalamic-pituitary-ovarian axis.

## Data availability statement

The original contributions presented in the study are included in the article/supplementary material. Further inquiries can be directed to the corresponding author.

## Ethics statement

The studies involving human participants were reviewed and approved by the Ethics Committee of Shengjing Hospital of China Medical University. The patients/participants provided their written informed consent to participate in this study.

## Author contributions

CX and BH contributed to conception and design of the study. HZ organized the database. JZ performed the statistical analysis. CX wrote the first draft of the manuscript. All authors contributed to manuscript revision, read, and approved the submitted version.

## Funding

The research was funded by Science and Technology Department People’s Livelihood Science and Technology Joint Program Funding of Liaoning Province (No. 2021JH2/10300125).

## Acknowledgments

Thanks to Editage for assisting with language editing services.

## Conflict of interest

The authors declare that the research was conducted in the absence of any commercial or financial relationships that could be construed as a potential conflict of interest.

## Publisher’s note

All claims expressed in this article are solely those of the authors and do not necessarily represent those of their affiliated organizations, or those of the publisher, the editors and the reviewers. Any product that may be evaluated in this article, or claim that may be made by its manufacturer, is not guaranteed or endorsed by the publisher.
